# Effect of playing position and microcycle days on the acceleration speed profile of elite football players

**DOI:** 10.1038/s41598-022-23790-w

**Published:** 2022-11-10

**Authors:** Antonio Alonso-Callejo, Jorge García-Unanue, Andrés Perez-Guerra, David Gomez, Javier Sánchez-Sánchez, Leonor Gallardo, Jose María Oliva-Lozano, Jose Luis Felipe

**Affiliations:** 1 IGOID Research Group, Department of Physical Activity and Sport Sciences, University of Castilla-La Mancha, Toledo, Spain; 2 Performance Analysis Department, UD Las Palmas, Las Palmas de Gran Canaria, Spain; 3 School of Sport Sciences, Universidad Europea de Madrid, C. Tajo, S/N, 28670 Villaviciosa de Odón, Madrid, Spain; 4 Health Research Centre, University of Almería, Almería, Spain; 5 Unión Deportiva Almería, Almería, Spain

**Keywords:** Computational biology and bioinformatics, Physiology

## Abstract

The aim of this study was to analyse the differences in the A–S profile of elite football players induced by playing position and the microcycle day. Players belonged to a second division club in the Spanish La Liga competition. They were classified into five playing positions: central defenders (CD), full backs (FB), midfielders (MF), wide midfielders (WMF) and forwards (FW). Microcycle days were categorised according to the days until matchday (MD, MD-1, MD-2, MD-3, MD-4 and MD-5). Data was collected along six microcycles, including one match per microcycle. The variables analysed were: maximal theoretical acceleration (A_0_), maximal theoretical speed (S_0_), maximal acceleration (ACC_max_), maximal speed (S_max_) and A–S slope (AS_slope_). Significant differences were found within positions and microcycle day for all variables (*p* < 0.05). Match day (MD) showed greater values than the training sessions in A_0_, ACC_max_ and S_max_ (*p* < 0.05). The highest values for variables associated with acceleration capabilities were found in CD on MD, whereas speed variables were higher in WMF. MD-2 showed the lowest values in all variables except for AS_slope_. Maximal acceleration and sprint abilities are therefore affected by playing position. Wide positions showed the highest speed capacity, and CD presented a likely acceleration profile. Higher values for all variables concerning the microcycle day, were achieved on MD, and were not reproduced during training with the consequent injury risk and performance decrease it takes.

## Introduction

The use of global positioning system (GPS) devices as method of monitoring workloads has increased exponentially in recent years^1^. These devices allow to understand the physical demands of sports in depth, according to an athlete’s age, tactical position or others contextual factors, and this enable performance analyst to establish individual profiles^2^. GPS devices are an effective and validated tool, with enough sensitivity to appreciate speed changes, and to analyse the physical demands^3^ and movement patterns^4^ in elite football. Analysts of elite football competitions have increased their observation of matches in order to examine the patterns and movements performed by players^5^.

Football is an intermittent team sport, in which high intensity efforts (10%) are combined with longer periods of low intensity^6^, however, both the distance and number of high intensity and sprint actions have increased (~ 35%) in recent seasons in elite football^7,8^. The most common action preceding a goal or an attempt to score a goal, is sprinting^9,10^. Power abilities such as the ability to accelerate are essential in developing other critical skills (e.g., jumping and changing of direction)^11^.

Previous investigations have suggested the use of the horizontal force–velocity (F–V) profile in order to gain a better understanding of sprinting and power skills^12,13^. The F–V profile is a linear regression of two axes (X axis = velocity and Y axis = force) developed by plotting the force applied in each speed, as the more speed is achieved the lower the force that can be applied. Although the force–velocity profile is usually measured through a single linear and field test with good validity and reliability^14,15^ it requires preparation and organisation, and it is not specific to team sports actions^14^. Morin et al.^14^ thus designed a new method known as the acceleration-speed (A–S) profile, based on in-situ data collection. This means that the data does not need to be collected through a specific test since the raw acceleration and speed data collected during training or a match is enough. Specifically, the A–S profile allows an understanding of the acceleration requirements in the whole velocity spectrum, and vice versa. For example, acceleration is greater when at lower velocities^14,16^. A regression model was designed in order to relate the initial speed and maximal individual acceleration^16^. The A–S profile is considered as a method for evaluating sprinting and acceleration abilities and not as workload indicator as sprint distance and acceleration distance can be. It is similar to the F–V profile and it allows the theoretical maximal speed and accelerations to be extrapolated as the individual’s sprint maximal capacity^14^. It has shown good reliability when evaluating elite football players^14^. Similar results correlate V_0_ and F_0_ (F–V profile variables) with the maximal theoretical speed (S_0_) and the maximal theoretical acceleration (A_0_) (A–S profile variables) respectively, representing the same mechanical concept^14^.

The F–V profile of athletes is variable depending on the sport and on the individual capabilities^17^. It has also been observed that acceleration- and sprint-related variables (e.g., maximal speed or accelerations) vary according to contextual variables such as the playing position. For example, wide positions involve higher speed and acceleration abilities^18^. Not only the playing position but also the microcycle load periodisation in elite football provokes oscillations in the workload variables between training sessions and matchday (MD)^19,20^. Commonly, Days 4 (MD-4) and 3 (MD-3) before the MD, the load is higher than Days 2 (MD-2) and 1 (MD-1) in which load progressively decreases^20^.

The aim of this study was thus to analyse the variability of the A–S profile of elite football players according to playing position and the microcycle day. The hypothesis is that training sessions MD-4 and MD-3 will present similar values to MD, and that MD-2 will show the lowest values in the microcycle. Wing positions such as fullback (FB) and wide midfielders (WMF) are expected to show higher speed and acceleration capabilities, and the lowest values will be found in midfielders (MF) and the central defender (CD).

## Materials and methods

### Experimental approach to the problem

An observational retrospective study was used to observe A–S profiles during six consecutive microcycles for elite male football players: Microcycle 1 (M1: from September 7th to 13th, 2021), Microcycle 2 (M2: from September 14th to 21st, 2021), Microcycle 3 (M3: from September 22nd to 26th 2021), Microcycle 4 (M4: September from 28th to October 4th, 2021), Microcycle 5 (M5: from October 5th to 10th, 2021), and Microcycle 6 (M6: from October 11th to 17th, 2021). There were no interventions further from the regular training and competition in the observational timeframe in this study. The Clinical Research Ethics Committee of the Castilla-La Mancha Health Service [Spain] approved this study based on the latest version of the Declaration of Helsinki (Ref.: 489/24022020).

### Subjects

A total of 25 male elite football players (age 25.16 ± 3.68 years old; body mass 75.08 ± 5.99 kg; height 178.96 ± 4.81 cm; body fat 10.2 ± 1.22%; VO_2peak_ 51.12 ± 3.57 ml/min/kg) agreed to participate in this study. The players were members of the first team of a professional Spanish club competing in La Liga SmartBank (Spanish second division). Players were grouped by playing position according to the tactical disposition of the team (1-4-4-2): five central defenders (CD), five forwards (FW), six wide midfielders (WMF), three full-backs (FB) and six midfielders (MF). There were no changes in playing positions along the six matches observed so positions were fixed at the beginning of the study and maintained until the end. Goalkeepers were excluded due to the different capabilities required by this position. Participants were informed about the study aims and procedures, and signed a written informed consent form before beginning the study.

### Procedures

The data was collected via global positioning system (WIMU PRO™, RealTrack System SL, Almería, Spain), with a sampling data rate of 18 Hz. These devices were previously validated as a reliable tool with which to collect physical data during football specific activities^3^. They have also been approved by the FIFA Quality Programme for the collection of velocity and positioning data^21,22^. Each player wore padded neoprene between the shoulder blade, where the device was attached. The data was analysed immediately after each training session and match using SPRO software v. 958 (RealTrack System SL, Almería, Spain).

A total of 31 sessions (25 training sessions and 6 competitive matches (MD)) were analysed. Training sessions were categorised as MD-1 (1 day before the match), MD-2 (2 days before the match), MD-3 (3 days before the match), MD-4 (4 days before the match) and MD-5 (5 days before the match). Match data only included players who participated for at least 60 min in the match as this was considered the minimal time required to achieve the maximal profile. The A–S profile variables (Table 1) were obtained for each session and player from a dataset that included the speed performed in each acceleration during the session. The aim of an A–S profile is to identify the linear regression of the maximal acceleration produced for any speed^14^. The A–S profile was plotted following the instructions given by Morin, et al.^14^. The minimal speed considered for starting the analysis was 3 m/s, because accelerations below this value are not considered maximal. Subintervals of 0.2 m/s of the speed data were set from 3 m/s to the maximal speed reached in the session (e.g., 3.0 to 3.2 m/s, 3.2 to 3.4 m/s etc.). The two maximal values for accelerations in each subinterval were identified. The linear regression was therefore fitted with the two maximal accelerations for each speed subinterval.

**Table 1:** Variables of the acceleration-speed profile.

Variable	Definition
A_0_	Maximal theoretical acceleration (ordinate axis intercept (y) in A–S linear regression)
S_0_	Maximal theoretical speed (abscissa axis intercept (x) in A–S linear regression)
AS_slope_	Linear slope. Calculated: -A_0_/S_0_
ACC_max_	Maximal acceleration (m/s^2^)
S_max_	Maximal speed (m/s)

The data used to set this linear regression was filtered using RStudio (version 3.6.0^©^ 2009–2021 RStudio, PBC) for each player and session (~ 60 raw data points). This software has been previously used in football investigations^23^. The A–S profile represented a linear regression in which acceleration was the ordinate axis and speed the abscissa axis, and it was fitted to these speed-acceleration points. After fitting, the residuals were analysed and outlier points were removed when out of a 95% confidence interval upper and lower limits around the linear function in order to improve the linear regression fitting and the overall accuracy of the model variables.

### Statistical analysis

Kolmogorov–Smirnov and Levene's tests were used to test the normality distribution and the homogeneity of variance. The results showed that data was normally distributed and displayed homogeneous variance (the Kolmogorov–Smirnov result varied from 0.093 to 0.235; p value from 0.047 to 0.200 and Levene's test varied from 1.090 to 2.106; p value from 0.079 to 0.361). The same player has several observations (several microcycles) in the dataset. Therefore, linear mixed models were used, which is a statistical method previously used in this type of analysis^24^. This statistic adjusts for correlation due to repeated observations on each subject over the different microcycles.

Firsltly, the variables in relative terms were first compared between different sessions related to specific training or match days in the microcycle (MD-1, MD-2, MD-3, MD-4 and MD-5) using linear mixed models, Microcycle was introduced as fixed effect and Player ID was introduced and modelled as a random effect.

Secondly, two-way linear mixed models were used to compare the variables in absolute terms between the different sessions (MD-1, MD-2, MD-3, MD-4, MD-5 and MD) and playing position (forward, FW; central-defender, CD; wide midfielder, WMF; full-back, FB; and midfielder, MF). Microcycle and playing position wers introduced as fixed effect and Player ID was introduced and modelled as a random effect.

The confidence level was established at 95%, and values of *p* < 0.05 were considered statistically significant. Differences were also studied using the standardised effect size differences (ES), and classified as negligible (ES < 0.2), small (ES between 0.2 and 0.6), moderate (ES between 0.6 and 1.2) and large (ES > 1.2). IBM SPSS Statistics version 25.0 software for Windows (SPSS Inc., IL, USA) was used for statistical analysis.

## Results

**Figure 1:**
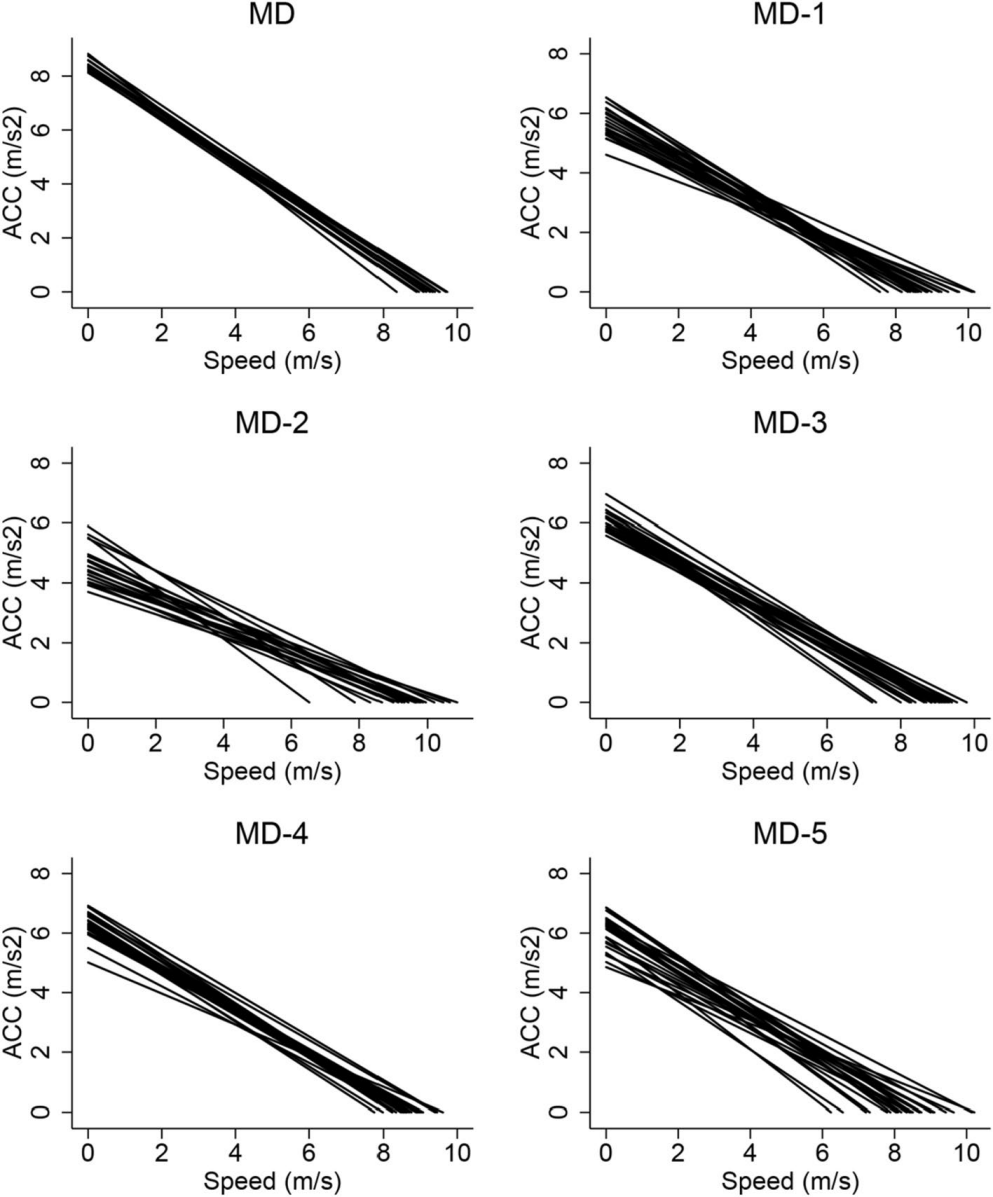
Acceleration-speed profile of elite football players on different training and match days in the microcycle. Acc = acceleration (m/s2); Speed (m/s). MD = matchday.

Significant differences were found between A–S profiles depending on the MD session and the different players included in the analysis (*p* < 0.05; Fig. 1).

**Figure 2:**
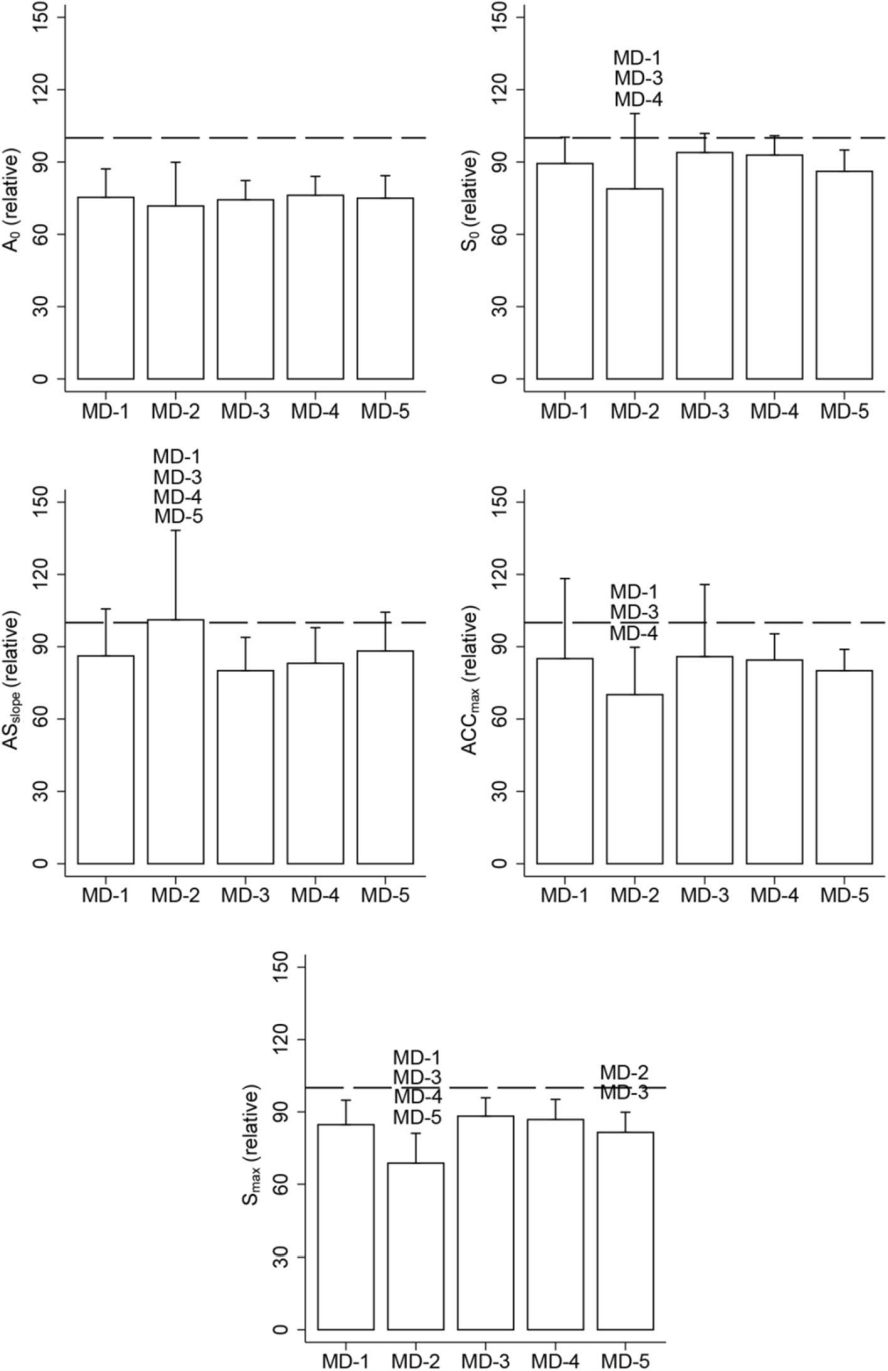
Variables in acceleration-speed profile on training sessions relative to match day. Data is presented as the percentage relative to matchday. A0 = theoretical maximal acceleration. S0 = theoretical maximal speed. Slope = -A0/S0. AccMax = maximal acceleration with an initial speed above 3 m/s. SMax = maximal speed. MD = match day.

The analysis of variance revealed significant differences between training session days in variables such as S_0_, AS_slope_, ACC_max_ and S_max_ at their relative match values (*p* < 0.05; Fig. 2)_._ S_0_ was lower in MD-2 in comparison to MD-1 (− 10.5%; 95% CI − 17.6 to − 3.43%; *p* < 0.05; ES = 0.5), MD-3 (− 15%; 95% CI − 22.1 to 8%; *p* < 0.05; ES = 0.8) and MD-4 (− 14%; 95% CI − 6,1 to 21.9; *p* < 0.05; ES = 0.7). AS_slope_ on MD-2 was significantly higher than MD-1 (15%; 95% CI 5.51 to 24.44%; *p* < 0.05; ES = 0.5), MD-3 (21.1%; 95% CI 11.6 to 30.5%; *p* < 0.05; ES = 0.8), MD-4 (18%; 95% CI 7.51 to 28.53%; *p* < 0.05; ES = 0.7) and MD-5 (12.9%; 95%CI 0.21 to 25.63%; *p* < 0.05; ES = 0.5). ACC_max_ was lower in MD-2 compared to MD-1 (− 14.9%; 95% CI − 25.05 to − 5.71%; *p* < 0.05; ES = 0.6), MD-3 (− 15.8%; 95% CI − 25.84 to − 5.71%; *p* < 0.05; ES = 0.6) and MD-4 (− 14.3%; 95% CI − 25.58 to − 3.1%; *p* < 0.05; ES = 0.9). S_max_ was lower in MD-2 than MD-1 (− 15.95%; 95% CI − 19.93 to − 11.98%; *p* < 0.05; ES = 1.3), MD-3 (− 19.5%; 95% CI − 23.44 to − 15.54%; *p* < 0.05; ES = 1.6), MD-4 (− 18.1%; 95% CI − 13.66 to 22.49%; *p* < 0.05; ES = 1.7) and MD-5 (− 12.8%; 95% CI − 18.11 to − 7.43%; *p* < 0.05; ES = 1.2), but MD-5 also showed lower values than MD-3 (− 6.7%; 95% CI − 12.05 to − 1.4%; *p* < 0.05; ES = 0.6).

Table 2 shows the A–S profile variable values by playing position and day of the microcycle. MD values were significant higher (*p* < 0.05) in all positions compared to the training days for A_0_, Acc_max_ and S_max_. MD-2 values were significantly lower than the rest of the days for ACC_max_ and S_max._

**Table 2:** Acceleration-Speed profile variables values by playing position and microcycle day.

		FW (1)	CD (2)	WMF (3)	FB (4)	MF (5)	
A_0_	MD-1	5.76 ± 1.01*	6.23 ± 0.85*	6.32 ± 0.88*	6.10 ± 0.78*	5.94 ± 1.03*	
	MD-2	5.80 ± 1.64*	7.27 ± 4.88^1,3,4,5^*	6.17 ± 1.17*	5.73 ± 1.13*	6.15 ± 2.46*	
	MD-3	6.18 ± 0.72*	6.26 ± 0.70*	6.15 ± 0.59*	6.23 ± 0.46*	6.19 ± 0.61*	
	MD-4	6.08 ± 0.61*	6.37 ± 0.55*	6.62 ± 0.67*	6.22 ± 0.36*	6.16 ± 1.23*	
	MD-5	5.94 ± 0.81*	6.18 ± 0.40*	6.17 ± 0.79*	6.25 ± 0.75*	6.04 ± 0.86*	
	MD	8.31 ± 0.36	8.68 ± 0.63	8.34 ± 0.40	8.46 ± 0.64	8.26 ± 0.81	
S_0_	MD-1	8.82 ± 2.00	8.15 ± 1.24	8.52 ± 0.85	8.69 ± 0.85	8.36 ± 0.79	
	MD-2	7.49 ± 3.64^5^*	7.27 ± 1.97^5^*	7.72 ± 1.37^5^*	7.82 ± 1.51^5^	5.66 ± 1.35*	
	MD-3	8.65 ± 0.79	8.63 ± 0.88	9.03 ± 0.75	9.04 ± 0.65	8.20 ± 0.89	
	MD-4	8.74 ± 0.63	8.80 ± 0.68	8.69 ± 0.67	8.84 ± 0.48	8.13 ± 0.99	
	MD-5	8.53 ± 1.13	8.50 ± 1.16	8.38 ± 0.86	8.45 ± 0.67	7.77 ± 0.60	
	MD	9.34 ± 0.52	9.34 ± 0.42	9.50 ± 0.34	9.22 ± 0.32	9.30 ± 0.85	
ACC_max_	MD-1	3.90 ± 0.57*	4.07 ± 0.50*	4.30 ± 0.59^1,5^*	4.11 ± 0.54*	3.96 ± 0.53*	
	MD-2	3.28 ± 0.76*	3.63 ± 1.00^1,5^*	3.93 ± 0.72^1,5^*	3.80 ± 0.74^1,5^*	3.27 ± 1.11*	
	MD-3	4.05 ± 0.43*	4.20 ± 0.53*	4.33 ± 0.36*	4.37 ± 0.36*	4.08 ± 0.38*	
	MD-4	4.23 ± 0.83*	4.32 ± 0.47*	4.45 ± 0.40^5^*	4.38 ± 0.74*	4.01 ± 0.89^3^*	
	MD-5	4.05 ± 0.35*	4.09 ± 0.66*	4.17 ± 0.59*	4.27 ± 0.31*	3.73 ± 0.45*	
	MD	5.10 ± 0.26	5.35 ± 0.48^3^	4.78 ± 1.18	5.24 ± 0.44	5.10 ± 0.32	
S_max_	MD-1	7.22 ± 0.66*	6.99 ± 0.94*	7.24 ± 0.66*	7.45 ± 0.69*	7.25 ± 0.57*	
	MD-2	5.84 ± 1.25*	5.96 ± 1.43*	6.38 ± 1.13^1,5^*	6.56 ± 1.15^1,2,5^	5.67 ± 1.56*	
	MD-3	7.25 ± 0.70*	7.32 ± 0.67*	7.71 ± 0.601^1,5^*	7.84 ± 0.52^1,5^	6.95 ± 0.71*	
	MD-4	7.32 ± 0.55*	7.32 ± 0.47*	7.43 ± 0.59*	7.67 ± 0.48^5^*	7.03 ± 0.87^4^*	
	MD-5	7.39 ± 0.79*	7.06 ± 1.02*	7.15 ± 0.71*	7.43 ± 0.44*	6.85 ± 0.58*	
	MD	8.35 ± 0.66	8.48 ± 0.40	8.82 ± 0.41^**5**^	8.47 ± 0.37	7.96 ± 0.57	
AS_slope_	MD-1	− 0.68 ± 0.17	− 0.79 ± 0.18	− 0.76 ± 0.16	− 0.71 ± 0.15	− 0.72 ± 0.18	
	MD-2	− 0.90 ± 0.44^2^	− 1.28 ± 1.79*	− 0.83 ± 0.25^2,5^	− 0.77 ± 0.24^2,5^	− 1.06 ± 0.82	
	MD-3	− 0.7 ± 0.12	− 0.74 ± 0.13	− 0.69 ± 0.11	− 0.70 ± 0.09	− 0.77 ± 0.16	
	MD-4	− 0.70 ± 0.09	− 0.73 ± 0.10	− 0.77 ± 0.14	− 0.71 ± 0.07	− 0.76 ± 0.16	
	MD-5	− 0.72 ± 0.19	− 0.74 ± 0.10	− 0.74 ± 0.09	− 0.75 ± 0.14	− 0.79 ± 0.16	
	MD	− 0.89 ± 0.08	− 0.93 ± 0.09	− 0.88 ± 0.05	− 0.92 ± 0.10	− 0.90 ± 0.15	

Variables: *A*_*0*_ theoretical maximal acceleration, *S*_*0*_ theoretical maximal speed, *AS*_*slope*_ − A_0_/S_0_, *ACC*_*max*_ maximal acceleration with an initial speed above 3 m/s and *S*_*max*_ maximal speed. *MD* matchday. *FW* forward, *CD* central defender, *WMF* wide-midfielder, *FB* full-back and *MF* midfielder. *Differences with MD (*p* < 0.05). ^1^Differences with FW (*p* < 0.05). ^2^Differences with CD (< 0.05) ^3^Differences with WMF (*p* < 0.05). ^4^Differences with FB (*p* < 0.05). ^5^Differences with MF (*p* < 0.05).

The results of the within positions analysis revealed substantially greater A_0_ in CD for MD-2 than WMF (1.09 m/s^2^; 95% CI 0.35 to 1.8 m/s^2^; *p* < 0.05; ES = 0.5), FW (1.46 m/s^2^; 95% CI 0.7 to 2.1 m/s^2^; *p* < 0.05; ES = 0.4), FB (1.54 m/s^2^; 95% CI 0.66 to 2.4 m/s^2^; *p* < 0,05; ES = 0.5) and MF (1.12 m/s^2^; 95% CI 0.36 to 1.87 m/s^2^; *p* < 0.05; ES = 0.3).

Differences between positions were also observed in MD-2, for S_0_, in which MF values were lower when compared to FW (− 1.83 m/s; 95% CI − 2.9 to − 0.75 m/s; *p* < 0,05; ES = 0.3), CD (− 1.61 m/s; 95% CI − 2.73 to − 0.5; *p* < 0,05; ES = 0.3), WMF (− 2.1 m/s; 95% CI − 3.11 to − 1.01 m/s; *p* < 0,05; ES = 0.5) and FB (− 2.16 m/s; 95% CI − 3.4 to − 0.91 m/s; *p* < 0,05; ES = 0.5).

The results concerning ACC_max_ showed greater values on MD-1 in WMF than FW (3.9 ± 0.57 m/s^2^; 95% CI *p* < 0.05; ES = 0.7) and MF (3.96 ± 0.53 m/s^2^; 95% CI *p* < 0.05; ES = 0.6). Inter-positions differences were also found on MD-2, with the Acc_max_ being lower in MF than in CD (− 0.36 m/s^2^; 95% CI − 0.7 to − 0.02 m/s^2^; *p* < 0.05; ES = 0.34), WMF (− 0.66 m/s^2^; 95% CI − 0.98 to  − 0.34 m/s^2^; *p* < 0.05; ES = 0.7) and FB (− 0.54 m/s^2^; 95% CI − 0.9 to − 0.17 m/s^2^; *p* < 0.05; ES = 0.6). Differences in MD for ACC_max_ were found within CD and WMF (− 0.57 m/s^2^; 95% CI − 1.13 to − 0.01 m/s^2^; *p* < 0.05; ES = 0.7).

There were significant differences (*p* < 0.05) for S_max_ between playing positions in MD-2, MD-3, MD-4 and MD. Lower values were found on MD-2 compared FW to WMF (-5.39 m/s; 95% CI − 0.97 to − 0.11 m/s; *p* < 0.05; ES = 0.5) and FB (− 0.71 m/s; 95% CI − 1.22 to 0.21 m/s; *p* < 0.05; ES = 0.6). On MD-2, FB therefore achieved greater values than CD (0.6 m/s; 95% CI 0.08 to 1.11 m/s; *p* < 0.05; ES = 0.5) and MF (0.89; 95% CI 0.38 to 1.38 m/s; *p* < 0.05; ES = 0.7). On MD-3, WMF reached greater S_max_ than FW (0.4 m/s; 95% CI − 0.8 to 0.86 m/s; *p* < 0.05; ES = 0.7) and MF (0.76 m/s; 95% CI 0.31 to 1.19 m/s; *p* < 0.05; ES = 1.1). The S_max_ values reached by FB, were also higher than FW (0.6 m/s; 95% CI 0.05 to 1.1 m/s; *p* < 0.05; ES = 1) and MF (0.89 m/s; 95% CI 0.4 to 1.41 m/s; *p* < 0.05; ES = 1.4) on MD-3. MF achieved lower S_max_ on MD than WMF (− 0.9 m/s; 95% CI − 1.6 to − 0.1 m/s; *p* < 0.05; ES = 1.8).

Significant results were found on MD-2 for the AS_slope_, in which CD showed lower values than FW (− 0.38; 95% CI − 0.61 to − 0.14; *p* < 0.05; ES = 0.3), WMF (− 0.45; 95% CI − 0.68 to − 0.21; *p* < 0.05; ES = 0.4) and FB (− 0.51; 95% CI − 0.78 to − 0.21; *p* < 0.05; ES = 0.5).

## Discussion

This study analysed the A–S profile of elite football players according to different playing positions, training days and matches. To the authors’ knowledge, this is the first study reported in the literature to conduct a longitudinal 6-weeks observation of this profile. The longitudinal observation offered reliable results, in which differences between microcycle session days and within playing position were found for all included variables of the A–S profile.

Previous investigations have described the A–S profile in two non-consecutive microcycles^14^, showing mean values without categorising by playing position or microcycle day. Reference values offered by Morin, et al.^14^ are 9.47 ± 0.52 m/s and 7.2 ± 0.4 m/s^2^ for S_0_ and A_0_ respectively in a whole microcycle. Our study, however, showed that S_0_ and A_0_ depends on playing position and microcycle day, showing a range from 5.66 ± 1.35 m/s (MF on MD-2) to 9.5 ± 0.34 m/s (WMF on MD) for S_0_ and 5.73 ± 1.13 m/s^2^ (FB on MD-2) to 8.68 ± 0.63 m/s^2^ (CD on MD) for A_0_. The S_0_ range values on MD in this study are also similar to the V_0_ values analysed in linear tests of the force–velocity profile by previous studies: 9.25 ± 0.46 m/s^12^, 9.25 ± 0.61 m/s^25^ and 9.2 ± 0.4 m/s^26^, when single sprinting F–V profile of elite football players was analysed. A_0_ values found in this study are similar to F_0_ values reported in the literature: 7.14 ± 0.58^12^, 7.14 ± 0.58^25^ and 8.4 ± 0.5 m/s^2^^26^. An A–S profile gives more specific data because it is obtained from a huge number of on-field football actions, however, more investigations are needed to firmly establish a correlation between A–S and F–V profiles. If both profiles were statistically correlated, the A–S profile could be applied with the applications shown by the F–V profile, such as fatigue and injury management^15,27,28^.

Previous studies have already shown significant differences between playing positions concerning ACC_max_ and S_max_^4,12,18^. The highest S_max_ was reached by WMF on MD (8.82 ± 0.41 m/s) and the lowest by MF (7.96 ± 0.57). Similar results to those in our study were found in other research with a team from the same division, in which WMF reached the greatest S_max_ (8.88 ± 0.44 m/s) and MF the lowest (7.91 ± 0.47 m/s) on MD^18^. Physical requirements are specific for each playing position^19,29^ and players develop their profiles according to those positional demands, what may explain the variability in the A–S profile variables according to playing position found in this study. For example, external players such as the FB and WMF cover greater distances sprinting than the other positions^30^ due to their tactical role. In consequence, they might express higher speed capacities in their position. The A–S profile may help coaches to select players with better capacities for the tactical role wanted.

Although more investigations are required, the continual evaluation offered by the A–S profile allows specific training to be prescribed, not only according to the day of the microcycle, but also according to playing position. The A–S profile could detect individual imbalances in acceleration and speed capabilities. It could be interesting to determine the optimal A_0_ and S_0_ for elite football players in order to set specific and individualised training programmes^17,31–33^ according to playing position and individual capabilities, and improve a specific range of the A–S spectrum. For example, the greatest values for S_max_ on MD were found in WMFs, who reached the lowest ACC_max_. In this case, specific training should be programmed for WMFs in order to improve the ACC_max_, with very heavy sled sessions^33,34^. On the other hand, positions with the lowest speed in both theoretical and maximal values should choose light loads (< 10% body mass) with the aim of improving the right side of the A–S profile, which corresponds to maximal velocity^35^.

More significant differences were found for ACC_max_ and S_max_ than for A_0_ and S_0_. This could be explained because A_0_ and S_0_ are theoretical values, and their accuracy depends on the R^2^ coefficient. A higher number of significant differences were found on MD-2, with the lowest values for acceleration and speed variables. This confirms the workload periodisation used by many strength and conditioning coaches in which, the two days prior to MD begin a tapering period in which the training load significantly decreases^36,37^. The training programme of this team establishes MD-2 as the day with the minimal workload in the microcycle, and uses this day for training strategy.

This research has some limitations that need to be considered. Football is an intermittent sport in which the physical demands oscillate, depending on the style of playing and training, the team formation, the individual characteristics of players in the same positions, and the match results^30,38,39^. This study included one team from a specific country, with a particular playing style and level within the league. Future research should include more teams from different competitions in order to provide results that could be extrapolated to different elite teams. Other contextual factors such as the results, or opponent levels, were not analysed. Only players who had played for at least 60 min were included in MD observations, so not all players participated on this day.

This study provides a meaningful and novel description of the differences found in the A–S profiles of elite football players. The results show significant differences in the variables of A–S profiles analysed according to position and day of the microcycle. Players reached higher values on MD, which means it is the most demanding day, as found in previous research^20^. MD is supposed to be the day with the highest load volume of the microcycle, but it is also the day with the highest requirements concerning speed and acceleration. This means that players do not reproduce what it is required on MD during training, with the possible consequences of increasing injury risk and decreasing performance. It must also be noted that CD was found to be the position with higher values in acceleration variables, however, previous research showed WMF to be the position with the best acceleration ability^18^. This finding could suggest that the sprint and acceleration profiles of each position could be affected by the playing style.

The main practical application of this study are the acceleration and speed values of elite football players offered, showing the individual nature of the A–S profile, which was unknown until now. This can help sport scientists and fitness coaches to understand the variability of this profile, and to design sessions orientated according to the specific positional demands. The results might therefore enable the F–V and A–S profiles to be connected in the future considering the potential advantages of applying the A–S profile in elite contexts (e.g., “testing without testing”). Analysing A–S profiles means that workload could be prescribed precisely and individually for each player, focusing on the spectrum (acceleration or speed) of the profile that needs to be improved by prescribing individualized resistance training programmes. For example, both acceleration or speed deficits can be identified. Moreover, the A–S profile gives information about the physical orientation of a training session, and whether the MD demands are covered along the training week.

## Supplementary Information

Supplementary Information.

## Data Availability

All data generated or analysed during this study is included in this published article [and its supplementary information files].

## References

[1] Kupperman N, Hertel J (2020). Global positioning system–derived workload metrics and injury risk in team-based field sports: a systematic review. J. Athl. Train.

[2] Hennessy L, Jeffreys I (2018). The current use of GPS, its potential, and limitations in soccer. Strength Cond. J..

[3] Bastida Castillo A, Gómez Carmona CD, De la Cruz Sánchez E, Pino Ortega J (2018). Accuracy, intra-and inter-unit reliability, and comparison between GPS and UWB-based position-tracking systems used for time–motion analyses in soccer. Eur. J. Sport Sci..

[4] De Hoyo M (2017). Analysis of the acceleration profile according to initial speed and positional role in elite professional male soccer players. J. Sports Med. Phys. Fitness.

[5] Di Salvo V (2010). Sprinting analysis of elite soccer players during European Champions League and UEFA Cup matches. J. Sports Sci..

[6] Rampinini E, Coutts AJ, Castagna C, Sassi R, Impellizzeri FM (2007). Variation in top level soccer match performance. Int. J. Sports Med..

[7] Barnes C, Archer DT, Hogg B, Bush M, Bradley PS (2014). The evolution of physical and technical performance parameters in the English Premier League. Int. J. Sports Med..

[8] Pons E (2021). A Longitudinal exploration of match running performance during a football match in the Spanish La Liga: A four-season study. Int. J. Environ. Res. Public Health.

[9] Faude O, Koch T, Meyer T (2012). Straight sprinting is the most frequent action in goal situations in professional football. J. Sports Sci..

[10] Sweeting AJ, Cormack SJ, Morgan S, Aughey RJ (2017). When is a sprint a sprint? A review of the analysis of team-sport athlete activity profile. Front. Physiol..

[11] Cometti G, Maffiuletti NA, Pousson M, Chatard JC, Maffulli N (2001). Isokinetic strength and anaerobic power of elite, subelite and amateur French soccer players. Int. J. Sports Med..

[12] Jimenez-Reyes P (2020). Seasonal changes in the sprint acceleration force-velocity profile of elite male soccer players. J. Strength Cond. Res..

[13] Morin J-B, Samozino P (2016). Interpreting power-force-velocity profiles for individualized and specific training. Int. J. Sports Physiol. Perform..

[14] Morin J-B (2021). Individual acceleration-speed profile in-situ: A proof of concept in professional football players. J. Biomech..

[15] Mendiguchia J (2014). Progression of mechanical properties during on-field sprint running after returning to sports from a hamstring muscle injury in soccer players. Int. J. Sports Med..

[16] Sonderegger K, Tschopp M, Taube W (2016). The challenge of evaluating the intensity of short actions in soccer: A new methodological approach using percentage acceleration. PLoS ONE.

[17] Samozino P, Rejc E, Di Prampero PE, Belli A, Morin J-B (2012). Optimal force–velocity profile in ballistic Movements—Altius: Citius or Fortius?. Med. Sci. Sports Exerc..

[18] Oliva-Lozano JM, Fortes V, Krustrup P, Muyor JM (2020). Acceleration and sprint profiles of professional male football players in relation to playing position. PLoS ONE.

[19] Guerrero-Calderón B, Morcillo JA, Chena M, Castillo-Rodríguez A (2022). Comparison of training and match load between metabolic and running speed metrics of professional Spanish soccer players by playing position. Biol. Sport.

[20] Oliva-Lozano JM, Gómez-Carmona CD, Fortes V, Pino-Ortega J (2021). Effect of training day, match, and length of the microcycle on workload periodization in professional soccer players: A full-season study. Biol Sport.

[21] FIFA. FIFA Quality performance reports for EPTS. (FIFA, https://football-technology.fifa.com/en/media-tiles/fifa-quality-performance-reports-for-epts/, 2020).

[22] Oliva-Lozano JM, Muyor JM (2022). Understanding the FIFA quality performance reports for electronic performance and tracking systems: From science to practice. Sci. Med. Footb..

[23] Guitart M (2022). Use of GPS to measure external load and estimate the incidence of muscle injuries in men’s football: A novel descriptive study. PLoS ONE.

[24] Fernandez-Navarro J, Fradua L, Zubillaga A, McRobert AP (2018). Influence of contextual variables on styles of play in soccer. Int. J. Perform. Anal. Sport.

[25] Jiménez-Reyes P (2018). Relationship between vertical and horizontal force-velocity-power profiles in various sports and levels of practice. Peer J..

[26] Haugen TA, Breitschädel F, Seiler S (2020). Sprint mechanical properties in soccer players according to playing standard, position, age and sex. J. Sports Sci..

[27] Nagahara R, Morin J-B, Koido M (2016). Impairment of sprint mechanical properties in an actual soccer match: A pilot study. Int. J. Sports Physiol. Perform..

[28] Mendiguchia J (2016). Field monitoring of sprinting power–force–velocity profile before, during and after hamstring injury: Two case reports. J. Sports Sci..

[29] Lago-Peñas C, Rey E, Lago-Ballesteros J, Casáis L, Domínguez E (2011). The influence of a congested calendar on physical performance in elite soccer. J. Strength Cond. Res..

[30] Lago-Peñas C (2021). Do elite soccer players cover longer distance when losing? Differences between attackers and defenders. Int. J. Sports Sci. Coach.

[31] Jaric S, Markovic G (2009). Leg muscles design: The maximum dynamic output hypothesis. Med. Sci. Sports Exerc..

[32] Newton R (2011). Developing maximal neuromuscular power: Part 2—Training considerations for improving maximal power production. Sports Med..

[33] Lahti J (2020). Individual sprint force-velocity profile adaptations to in-season assisted and resisted velocity-based training in professional rugby. Sports.

[34] Morin J-B (2017). Very-heavy sled training for improving horizontal-force output in soccer players. Int. J. Sports Physiol. Perform..

[35] Petrakos G, Morin J-B, Egan B (2016). Resisted sled sprint training to improve sprint performance: a systematic review. Sports Med..

[36] Clemente FM (2019). Characterization of the weekly external load profile of professional soccer teams from Portugal and the Netherlands. J. Hum. Kinet..

[37] Martín-García A, Díaz AG, Bradley PS, Morera F, Casamichana D (2018). Quantification of a professional football team's external load using a microcycle structure. J. Strength Cond. Res..

[38] Aquino R (2017). Effects of competitive standard, team formation and playing position on match running performance of Brazilian professional soccer players. Int. J. Perform. Anal. Sport.

[39] Yi Q (2019). Technical and physical match performance of teams in the 2018 FIFA World Cup: Effects of two different playing styles. J. Sports Sci..

